# Visual Perception in Migraine: A Narrative Review

**DOI:** 10.3390/vision5020020

**Published:** 2021-04-28

**Authors:** Nouchine Hadjikhani, Maurice Vincent

**Affiliations:** 1 Martinos Center for Biomedical Imaging, Massachusetts General Hospital, Harvard Medical School, Boston, MA 02129, USA; 2 Gillberg Neuropsychiatry Centre, Sahlgrenska Academy, University of Gothenburg, 41119 Gothenburg, Sweden; 3 Eli Lilly and Company, Indianapolis, IN 46285, USA; maurice.vincent@me.com

**Keywords:** migraine aura, vision, Alice in Wonderland Syndrome

## Abstract

Migraine, the most frequent neurological ailment, affects visual processing during and between attacks. Most visual disturbances associated with migraine can be explained by increased neural hyperexcitability, as suggested by clinical, physiological and neuroimaging evidence. Here, we review how simple (e.g., patterns, color) visual functions can be affected in patients with migraine, describe the different complex manifestations of the so-called Alice in Wonderland Syndrome, and discuss how visual stimuli can trigger migraine attacks. We also reinforce the importance of a thorough, proactive examination of visual function in people with migraine.

## 1. Introduction

Vision consumes a substantial portion of brain processing in humans. Migraine, the most frequent neurological ailment, affects vision more than any other cerebral function, both during and between attacks. Visual experiences in patients with migraine vary vastly in nature, extent and intensity, suggesting that migraine affects the central nervous system (CNS) anatomically and functionally in many different ways, thereby disrupting several components of visual processing. Migraine visual symptoms are simple (positive or negative), or complex, which involve larger and more elaborate vision disturbances, such as the perception of fortification spectra and other illusions [1]. Based on the physiology of vision, migraine visual manifestations may serve as clues to a better understanding of the mechanisms underlying this intriguing condition. Here we provide a narrative review of visual manifestation associated with migraine.

## 2. The Excitable Migraine Brain: With Aura or without Aura, That Is the Question

It appears that susceptibility to migraine is related to an imbalance between excitatory and inhibitory systems. The origins of this imbalance have not been totally elucidated. Several hypotheses, however, can be put forward: (1) Abnormal function of ion channels—genes that have been associated with certain types of migraine, including the familial hemiplegic migraine CACNA1A, ATP1A2, and SCN1A genes [2], alter this balance in favor of increased excitability; (2) Abnormalities in the thalamus, which plays a major role in cortical excitability control [3]—in a study where we examined thalamus microstructure using a multiparametric approach, we showed microstructural differences in the lateroposterior and the pulvinar nuclei of patients with migraine compared with healthy control participants. Because both these thalamic nuclei are highly connected with visual striate and extrastriate cortices, these differences could be associated with an altered modulation of excitability in the visual cortex, facilitating the occurrence of cortical spreading depression (CSD) and visual aura. Finally, (3) sex hormones play a complex role in cortical excitability. It has been shown in an animal model that increased susceptibility to CSD is reduced after ovariectomy, casting light on the much higher prevalence of migraine in females compared to males [4].

Differences in visual processing outside of migraine attacks have been studied for many years, using different techniques such as the recording of electrical/magnetic activity (electro-encephalography, EEG with visual evoked potential (VEP) also known as event-related potential (ERP), and magneto-encephalography, MEG). The electrical signal, recorded at the scalp, is expressed as positive (P) or negative (N) inflections at a certain time. For example, N270 is a negative signal peaking at 270 milliseconds that is typically seen in the occipito-temporal region in response to the perception of faces. Different studies have, on the other hand, provoked activation or deactivation of the cortex using transcranial magnetic stimulation (TMS) and compared brain excitability between groups. They tend to suggest a hyperexcitable brain and habituation deficits. We list below a series of findings revealed by these different methods.

TMS, a non-invasive technique that uses magnetic fields to stimulate the brain, has been used to probe brain excitability. Aurora et al. [5] found that their patients who had migraine with aura (MWA) showed a lower threshold excitability over the occipital cortex compared to healthy control participants, a finding that was replicated by Mullener and colleagues [6]. Batelli et al. [7] applied their stimulations more anteriorly and laterally, and examined visual cortical excitability by stimulating over area MT/V5 (an area involved with motion processing) in their MWA and migraine without aura (MWoA) patients. They found that both migraine types had hyperexcitability over this region, compared to healthy control participants. More recently, Brigo et al. [7] performed a meta-analysis of the TMS literature in migraine that included ten trials. They reported that both MWA and MWoA had statistically lower phosphene thresholds compared to healthy control participants using a circular coil for the stimulation, further supporting the hypothesis of a hyperexcitable visual cortex in migraine.

Visual processing starts in the retina, where axons of the ganglion cells form the optic nerve and project to the thalamus (lateral geniculate nucleus (LGN) and pulvinar) and the superior colliculus. The LGN sends its projections to the primary visual cortex—or V1—where the information is arranged in columns of specific orientation, as described by Hubel and Wiesel at the end of the 1950s [8]. As information moves up in the visual system’s hierarchy, area V2 is organized to respond to contours, textures and location, and thereafter it is distributed into different attributes such as color, face perception, etc. The different visual phenomena in migraine are closely related to the organization and function of the visual system.

Electrical recordings (VEP/ERPs) have revealed abnormalities in the processing of patterns, color, and face processing, in people with migraine between attacks.

### 2.1. Patterns

Afra and colleagues [9] compared VEP during long periods (15 min) of repetitive pattern-reversal stimulation in MWA and MWoA patients interictally, as well as in healthy control participants. While habituation (i.e., a reduction in neural response to continuous stimuli) could be observed in healthy control participants, the signal remained stable in both groups of patients, showing an interictal habituation deficit in cortical information processing.

More recently, Fong et al. [10] used striped patterns of specific spatial frequencies (0.5, 3, and 13 cycles-per-degree) in MWA and MWoA subjects and healthy control participants. They found that people with migraine had a significantly increased N2 amplitude for stimuli with 13 cpd gratings and proposed that this is in support of the cortical hyperexcitation hypothesis in migraine.

### 2.2. Color

Color signals in V1 are treated in color cell clusters named blobs, which project to V2 where they form thin stripes before sending projections to the brain’s color selective area in the inferior occipito-temporal region, which also has a specific retinotopic organization and a distinct foveal representation [11]. Haigh et al. [12] compared the behavioral and neural responses to chromatic patterns of increasing separation in migraine groups with and without aura and in healthy control participants. In addition, they asked the participants to rate their level of discomfort during data acquisition. They found that while greater chromaticity separation increased neural excitation in both migraine and healthy control groups, only the migraine group rated gratings as being disproportionately uncomfortable, and they exhibited greater effects of chromaticity separation in ERP amplitude across occipital and parietal electrodes, consistent with a hyperexcitability of the visual system.

### 2.3. Face Processing

Face processing is a complex system that involves a network of brain areas, comprising the occipital face area (OFA) and the fusiform face area (FFA) (for review, see [13]). One of the neural signature of face processing is the N170 [14]. Akdeniz and colleagues [15] used ERP and measured the neural correlates underlying face and face pareidolia processing in migraine groups with and without aura and in a healthy control group, by examining the N170, Vertex Positive Potential (VPP) and N250 mean amplitudes and latencies, which were generally greater in the migraine groups. In line with the evidences discussed above, they concluded that patients with migraine demonstrate visual cortical hyperexcitability.

## 3. What Patients with Migraine See and Do Not See

The most remarkable migraine visual experiences are related to the aura (from the Greek αύρα—breeze). Aura consists of fully reversible neurological dysfunctions, isolated or in successive combinations, that develop gradually, typically before the headache phase of a migraine attack [16]. Beyond visual symptoms (the visual aura—VA—characterized by varying visual perceptions, such as scintillating zig-zag patterns), aura can include sensory and/or language disturbances in isolation or combined, but vision is the most frequent CNS dysfunction, present in 98–99% of the aura episodes [1,17]. Interestingly, “mental efficiency” has been reported as impaired during VA progression [18], suggesting a temporarily dysfunctional cortical processing during aura. Although migraine predominates in women, male and female MWA patients present aura to the same ~33–38% proportion. In a retrospective study including 952 patients, migraine auras lasted on average ~27 min. [19]. This is in accordance with a prospective analysis of 216 diary-recorded auras, in which the average duration was 30 min. [1] Queiroz et al. found retrospectively that 65.5% of specifically visual auras lasted 5 to 30 min [20].

The archetype of VA is the fortification spectra, or teichopsia (from the Greek “town wall” and “vision”, as coined by Airy in 1870) [20,21] which accounts for ca. 40% of the reports [20]. This phenomenon—so characteristic that it may be considered almost as pathognomonic of migraine—is characterized by a series of complex interlacing angulated black-and-white (less frequently colorful) flickering and scintillating lines and bars that start as a small greyish spot in the visual field, more often paracentrally, less frequently at the center [21,22], growing over time from near the center towards the periphery, leaving a scotoma behind.

In the majority of the cases the visual phenomenon appears at the visual field contralateral to the headache, which appears from 0 to 60 min after the VA [21]. According to Queiroz et al., the gap between the end of the VA and the headache onset is less than 30 min in 65% of the cases [20]. The VA can occur always at the same side of the visual field (33%), vary from side to side (26%), be always bilateral (36%), or sometimes unilateral and bilateral (5%) [1]. This abnormality, which initially impairs the vision at a certain region of the visual field, expands progressively for 20 min or so to the periphery, assuming a “c” or a horseshoe shape, edged by typically bright and dark zigzag lines with bars and corners [21,23]. The inner angle in this c-shaped serration pattern, close to its center, was estimated as ~45°, increasing progressively until ~70° at the periphery [24]. A bean-shaped area, characterized by low visual acuity inside this zig-zag phenomenon, follows the expanding visual scintillation.

Edward Hare, self-observing 12 of his fortification spectra attacks, reported that the change in diameter of the “spectral figures” increased with time logarithmically, while the rate of oscillation diminished [25]. Another carefully self-observed and detailed collection of 1000 VAs of the fortification spectra type drawn over 20 years by a 71-year-old male suffering from migraine provided important insights on the propagation of the aura phenomenon. According to this databank, attacks originated mostly within 10° eccentricity in the visual field, propagating first predominantly in the lower nasal fields and spreading to the upper temporal fields [26]. The VA progression from the center to the periphery is more frequent then the opposite [27].

Other simpler aura visual patterns, isolated or in combination, include flashes of bright light, phosphenes (small bright dots), white spots described as “falling stars”, focal visual field defects, hemianopia, foggy or blurred vision, “visual snow” [28], or dimness of vision [1,22]. Most aura symptoms present more than one of these symptoms, with blurred vision (25%) and bright flashes of light (25%) being the most frequent patterns [1].

Color changes have been reported less frequently in migraine. Colorful auras were identified by 40% of the subjects studied by Queiroz et al., 18.0% of them presenting VAs in color exclusively [20]. Apart from shorter duration (1–2 min), round and multicolored image perceptions have traditionally supported the diagnosis of visual partial epileptic seizures, contrary to angular, zig-zagged, frequently black-and-white longer perceptions, supposedly more frequent in migraine VA [29]. Colorful aura perceptions included rather small than large dots, lines, or the fortification spectrum [30,31]. In a retrospective study of VA in 122 patients with migraine, the patterns were described as black-and-white, black-and-silver, always colorful, both colorful and black-and-white, and without color in 30.3%, 20.5%, 18.0%, 22.2%, and 9.0%, respectively [20].

Photophobia, a sign of hyperexcitability, is one of the distinctive symptoms of migraine, including during the interictal phase [32]. Color perception changes and hypersensitivity have been reported in migraine, which theoretically could simply result from photophobia itself, or from a direct migraine effect on color processing [33]. Color perimetry has shown abnormal perceptions of red and blue in patients with migraine. In one study, the dysfunction in red perception was greater in MWA patients, who also present higher levels of photophobia [34]. During migraine attacks, green light reduced headache intensity in ~20% of the subjects, contrary to white, blue, amber, and red lights, which exacerbated the pain. According to the authors, these findings, associated with thalamic, visual evoked potentials, and electroretinographic studies suggest that photophobia and specific color aversions originate in the retina and thalamus rather than in cortical visual processing areas [35].

Color processing dysfunctions may be involved in migraine-induced visual misperceptions [36] as suggested by patients referring to dimmed colors/achromatopsia [37] or, more frequently, color exaggeration in general [38]. One of our patients claimed the red color becomes so aggressive that “it seems to attack me”. Others describe vision as “if someone presses the color saturation button in a TV remote all way up”.

### Complex Visual Manifestations and the Alice in Wonderland Syndrome

The psychiatrist John Todd wrote that, “While there is wide appreciation of the fact that epileptic subjects, and their blood relatives, are prone to experience bizarre disturbances of the body image, few realize that essentially similar disorders affect migraine subjects and their families. As a result, many of these patients are unjustifiably dubbed ‘neurotic’ and referred to a psychiatrist, while others torture themselves with secret misgivings concerning their sanity” (page 701 of [39]). To group these altered bizarre misperceptions in size, shape or proportions of patient’s bodies and other objects he proposed the expression “the syndrome of Alice in Wonderland” (AIWS), according to Lewis Carrol’s book Alice’s Adventures in Wonderland [39]. AIWS may occur as a manifestation of different diseases, such as migraine, epilepsy, infections, cerebrovascular, and psychiatric disorders [40]. Nonvisual AIWS phenomena and the occurrence in diseases other than migraine are beyond the scope of the present work.

AIWS, which most probably originates from dysfunctions at the temporo-parieto-occipital carrefour [41], involves different complex perceptions including the sensation of changes to the passage of time [42]. As illustrated by Sacks (pp. 94–95), “the term *cinematographic vision* denotes the nature of visual experience when the illusion of motion has been lost. At such times, the patient sees only a rapidly-flickering series of ‘stills’, as in a film run too slowly. The rate of flickering is of the same order as the scintillation-rate of migrainous scotomata or paresthesia (6 to 12 per second), but may accelerate during the course of the aura to restore the appearance of normal motion, or (in a particularly severe, delirious aura) the appearance of a continuously-modulated visual hallucination”. The same author described mosaic vision like “the fracture of the visual image into irregular, crystalline, polygonal facets, dovetailed together as in a mosaic” [43].

Anatomically close to the temporo-parieto-occipital carrefour is the inferior right parietal lobule. This cortical area in the nondominant hemisphere and its connections with temporal and ventrolateral frontal cortices have been implicated in unilateral spatial neglect [44,45]. Interestingly, unilateral spatial neglect was reported in two patients with sporadic hemiplegic migraine. First, a 13-year-old girl experienced flickering light and blurred vision to the left hemifield occurring before the onset of a contralateral occipital headache, which was accompanied by mild left hemiparesis, left hemihypesthesia, and dysarthria. She collided frequently with obstacles on her left side and had a positive left neglect drawing test. All symptoms disappeared in 24 h [46]. Second, a 20-year-old woman with migraine with typical visual aura since the age of 15: during one of her attacks, she presented short-lasting scintillations in the right visual field, followed by a right-sided headache (14 h duration), together with left-sided paresthesia and hemiparesis, both persisting for four hours. Drawing tests confirmed a left unilateral spatial neglect that disappeared with the resolution of the neurological symptoms [47]. We speculate that transient visual neglect and attention deficits may be part of migraine aura more often than hitherto supposed. These phenomena could be overlooked because of short duration, subtle and difficult to recognize or describe symptomatology, and lack of proactive search among physicians. In this context, it is remarkable that patients with migraine presented interictally lower BOLD-fMRI activation of the right temporal parietal junction on visual attention tasks [48], which is in line with interictal findings that patients perform worse than control subjects in visuospatial tasks with shift in attention to the right [49]. Other nonvisual cortical dysfunctions documented during migraine aura, such as apraxias, are not within the scope of this review.

As many as 42 different visual phenomena have been related to AIWS, in which the symptomatology is characterized by distortions of real sensory perceptions, contrary to hallucination, typically considered to be constructions independent from real stimuli [40]. Many of these complex visual symptoms have been recognized as part of possible migraine visual symptoms (Table 1). A likely mechanistic account links migraine sight dysfunctions to secondary and tertiary cortical vision processing areas and their connections.

In a prospective study, auras with visual perception disturbances were characterized exclusively by complex symptoms (37%), experienced also with either one positive or one negative symptom (54%), or both (9%) [1]. More recently, a prospective study targeting AIWS symptoms with an ad hoc questionnaire revealed that up to 19% of patients in a tertiary referral headache unit reported symptoms related to AIWS [50].

Among bizarre visual temporary dysfunctions associated with migraine is prosopagnosia, or the inability to distinguish human faces. Face identity recognition is a specialized neurological ability that plays a crucial surviving function in mammals. It involves a dedicated cortical circuitry that includes parts of the inferior occipital gyrus and the occipital and temporal fusiform gyrus [51]. A series of reports indicate that patients with migraine may present difficulties in recognizing other people’s faces to various degrees [37,38,52,53]. We have observed two patients who presented “hemiprosopagnosia” as part of their migraine attacks (unpublished). One of these patients described a transitory prosopagnosia as part of her migraine aura that was restricted to one side of people’s faces. She noticed this in other people, in her own image in a mirror, as well as in faces on a computer screen. The affected half face looked to her swirled, scrambled. Migraine-related prosopagnosia may be not restricted to MWA and perhaps is present in mild forms interictally, too [38,54].

**Table 1:** Migraine-related visual complex and unusual visual phenomena.

Abnormality	Reference
Lilliputian or Brobdingnagian vision (micropsia or macropsia): Objects or people perceived as too small or too large	[55]
Metamorphopsia: Objects or people are distorted, “*monstrous faces in others*” (MV), half of the observed face shifting upwards or downwards, or changes in lines or angles of the features of objects or faces	[1,56]
Misperceptions of body parts: segments of the body seen gigantically, transparent, perception of the body being split in two, vision of the hair growing quickly “*to cover all the floor*” (MV)	[55,56]
Teleopsia: Seeing objects as much farther. It may refer to walls, “*giving the impression of a much larger ambient, or just one object in particular*” (MV)	[1]
Pelopsia: Seeing objects as much nearer	[1]
Allesthesia: Objects are viewed inverted or at the opposite homonymous field	[55]
Polyopia: perception of objects or faces in many copies	[56]
Mononuclear diplopia	[57]
Tunnel vision	[22,30]
Prosopagnosia	[37,38,52,53]
Increased or decreased misperception in the rate of movement*:* “*book pages passing too quickly*”, “*lights in a tunnel succeeding in astonishing speed*” (MV)	[56]
Apparent movement of stationary objects	[56]
Waviness of linear contours	[56]
Objects with sharper contours, with exaggerated perspective or without a third dimension, looking diagrammatic	[43]
Corona phenomena: perception of colored or shining border around objects	[1,56]
Oscillopsia	[1]
Fragmented visual perception resembling “cracked glass” Kaleidoscope-like, mosaic vision	[43]
Impression of seeing through water heat waves, like “*looking at a distance close to the asphalt pavement in a very hot day*” (MV)	[1]
“Like a negative of film”	[22,30]
Complex hallucinations	[22,30]
Anopia—transient cortical blindness	[30]

MV: personal observations.

## 4. Confusing Terminology: Retinal Migraine, Ophthalmic Migraine, Ocular Migraine

Different terms have been used to address visual symptoms in migraine. Appropriate terminology is critical because inaccurate descriptions have clinical and pathophysiological implications. “Ophthalmic migraine”, “ocular migraine”, “anterior visual pathway migraine”, and “monocular migraine” are confusing and not listed in the current version of the international classification of headache disorders (ICHD-3) [16]. The appropriate name to describe migraine cases associated with aura characterized by visual symptoms is *migraine with aura*, coded as 1.2, or in case of a more specific diagnosis, *migraine with typical aura,* coded as 1.2.1. Aura is mechanistically a cortical phenomenon, retrochiasmatic in nature, affecting, therefore, both visual fields as homonymous phenomena. “Ophthalmic” or “ocular” are inappropriate terms in this context because they imply an origin related to the eye.

Retinal migraine (RM), which likewise is often mistakenly termed, is listed in the ICHD-3 under the code 1.2.4. To fulfill these diagnostic criteria, patients must present fully reversible, monocular, positive and/or negative visual phenomena during an attack confirmed by clinical visual field examination and/or the patient’s drawing of a monocular field defect. In practice, however, almost every patient tends to explain a homonymous visual field deficit as monocular. Physicians must confirm the nature of the visual defect by a careful history taking, particularly when dealing with patients with migraine. Because the distinction between a hemi-field deficit from a monocular visual deficit is particularly difficult, the physician must instruct the patient to alternately cover one and the opposite eye to address the putative unilaterality of the symptom.

The confirmation of RM diagnosis, which is characterized by monocular visual loss, is both difficult and rare, involving circulatory mechanisms. It is possible that the majority of RM cases published so far are not related to migraine at all [58]. The diagnostic confusion increases, for example, by RM reports without headache. In a review of 60 articles describing 142 patients with visual symptoms attributed to retinal migraine, 39 cases had had persistent visual loss because of central retinal artery occlusion (11 cases), cilioretinal artery occlusion (4 cases), focal retinal ischemia (1 case), central retinal vein occlusion (2 cases), ischemic optic neuropathy (6 cases), optic atrophy (5 cases), or no explanation (1 case). Among the 103 patients with transient visual loss considered as RM, only 16 had compatible clinical pictures. Furthermore, among transient monocular visual losses attributed to RM (or equivalent terms for this condition), 12 patients had segmental retinal vasospasm of arteries or veins, among which only one had headache, during or immediately after the visual loss, that did not satisfy the diagnostic criteria for migraine [59].

Other authors describe monocular teichopsias or spreading negative visual losses as blurring, “grey-outs”, and “black-outs”, leading partial (altitudinal, quadrantic, central, or arcuate) or complete blindness [60]. Once a visual deficit is confirmed as monocular, the differential diagnosis must involve causes of prechiasmatic lesions affecting the optic nerve, retina, blood vessels, or any tissue possibly involved with visual losses. After excluding all alternative possible causes, the ICHD-3 RM diagnostic criteria must be checked for final confirmation.

## 5. A Riddle behind Retinal Migraine

Spreading depression (SD), the cortical neurophysiological counterpart of migraine aura, has been observed in retinas in vitro. Isolated chick retina is a reliable, avascular, commonly studied retina SD model, in which the SD phenomenon changes the retina color, allowing visual observation of the slow SD propagation (typically 2–3 mm/min) [61,62]. Retinal SD has been addressed in other species, since the original observation in amphibians [63], including fish [64], mammals [65]—rat [66] and possibly cat [67]. One of the most important features of SD is its slow progression. Should retinal SD underlie the RM phenomenon, the monocular visual clinical deficit would probably mirror the SD slow spread and be characterized by an expanding, slowly progressing monocular visual deficit, edged by a scintillation or some kind of positive visual phenomenon. This is incompatible with RM cases described as sudden monocular total darkness [68].

In the case published by El Youssef et al. [69], the RM monocular changes are described as “seeing a curtain moving in nasal to temporal direction, disappearing gradually, followed by a left periorbital pulsatile headache lasting 10 min”, but details on the progression, its pace and duration are not provided. The ictal fundus showed multiple vasoconstrictions at the left central retina, which disappeared after attack resolution. No evidence of a “spreading” pattern was documented [69]. It is possible that retinal vasospasm provokes retinal SD which in turn leads to retinal migraine symptoms.

Documentation on spreading depression in human retinas and its possible relation with retinal migraine, either in conjunction with transitory retinal ischemic events or not, is lacking. RM remains as a very rare and controversial disorder, not to be confused with migraine with aura.

## 6. Visual Aura and Blindness

VA does not depend on visual input, as it can remain with one or both eyes closed [31]. Visual auras can also be seen with eyes opened in complete darkness. Self-observing one of his own occasional visual fortification spectra progressions in a complete dark environment manifested as a typical expanding black-and-white flickering zig-zag “C” shape (never suffered a migraine headache), one of the authors (MV) remarked that the black components appear vividly and as distinguished from the dark background as the white parts, suggesting that both black and white perceptions are positive phenomena of equal intensity.

Because the visual physiology is intimately involved with migraine expression, VA and photophobia have been addressed in visually impaired patients with migraine (VIPM). We investigated the presence of migraine and VA manifestations in blind adult patients [70]. In our series of 200 visually impaired patients, 63 (32.7%) fulfilled the International Classification of Diseases (ICD-10) code H54—bilateral blindness criteria corresponding to visual acuity impairment categories 3 (worse than 20/400 or 0.05; equal or better than 20/1200), 4 (worse than 5/300 or 0.02; equal or better than light perception), or 5 (no light perception). (WHO Study Group on the Prevention of Blindness, Geneva, 6–10 November l972, WHO Technical Report Series No. 518, 1973).

Among those, 23 (37%) presented migraine, 8 of whom with aura, among which 7 of the were VA type (one subject presented aura exclusively as auditory perceptions). All patients became blind after birth (from age 5 to 51. Migraine started before blindness in 6 patients, right after blindness in one, and much later in one—the subject with “auditory aura”). Three patients presented typical VA, contrary to four with atypical presentations. VA symptoms were atypical because of length (too short, i.e., less than 5 min according to ICHD-3 [16], color (blue, silver, or fire-like, contrasting to usual descriptions in migraine), and/or shape (round shapes) patterns. Among the six amaurotic patients with previous VA, four failed to present aura after the onset of blindness. In one, the VA remained clinically unchanged. In the last patient the aura perception modified as vision deteriorated, and eventually disappeared (before blindness: scintillations; after blindness: perception of “waves colored as fire” and “sparks crossing the air”). Interestingly, the patient who became blind by the age of five and started experiencing migraine during adolescence, reported an “auditory aura” perceived as an uncharacteristic noise and no visual phenomena. Auditory manifestations suggesting an aura-like phenomenon are rarely described in migraine [71]. Our “auditory aura” case, similarly to other observations in VIPMs [72], suggests that acoustic aura could be more frequent among blind subjects, possibly as a result of overactivation and/or reorganization of cortical areas related to hearing in blind subjects.

A constant normal visual input is probably necessary for the phenotypical construct of the migraine VA, as indicated by the fact that the majority of the aura phenomena disappeared and/or were atypical in our VIPM. Although the patients in our series did not report photophobia after blindness onset (present in all but one previous to vision loss), sensitivity to light has been documented in blind patients with migraine [73]. This discrepancy can be related to methodological issues or to the fact that photophobia in these patients may depend on the integrity of non-image forming visual pathways originating in melanopsin-rich retinal ganglion cells which converge on thalamic trigeminal sensory pathways that carry pain signals during migraine attacks [35]. Therefore, the integrity of the optic nerve is necessary for the presence of photophobia in blind people with migraine. Contrary to subjects with complete optic nerve damages, blind patients with intact optic nerves may present light-induced headache exacerbation [74].

Temporary binocular blindness can rarely be present in people with migraine, usually as a single, isolated, totally reversible episode, not related with other aura symptoms. The duration can vary from seconds to 2 h. The mechanisms underlying bilateral blindness during migraine attacks remain obscure [75].

## 7. Interictal Visual Symptoms

Migraine is usually conceived as a paroxysmal disease characterized by headache attacks separated by asymptomatic interictal phases. However, clinical, physiological, and neuroimaging data indicate that the interictal phase differs from healthy control subjets in many aspects, including vision. Sensory processing is significantly different in people with migraine, who are more sensitive to visual, acoustic, odoriferous, and somatosensorial stimuli [76].

Afterimages consist of physiological positive or negative visual perceptions that persist following visual stimulation, according to ON and OFF activity in visual receptive fields [77]. Afterimages in people with migraine are shorter interictally as compared to healthy control participants, increase progressively during the two days immediately prior to an attack and reach the maximum on the headache day [78]. This suggests changes in visual processing at different points during the migraine cycle. Transient and steady-state visual-evoked potentials suggest a particular imbalance between excitation and inhibition in the visual cortex that occurs interictally [79].

### 7.1. Photophobia

During migraine attacks, photophobia is usually defined as exacerbation of the headache secondary to ambient light exposure. Other components of photophobia outside migraine attacks include aversion to light, and attacks triggered by light. People with MWA and MWoA report more interictal aversion to light, have more photophobia symptoms (such as wearing sunglasses) and lower mean visual stress thresholds as measured by grating pattern induced unpleasantness (grating patterns that are bothersome, painful to look at, or irritating to the eyes) than control participants [80,81].

Migraine interictal photophobia may correlate with anxiety, depression and sleep disorders [82], possibly pointing to a higher severity within the migraine phenotype spectrum. Mechanistically, bright light enhances the activity of thalamic trigeminovascular neurons that transmit pain from the meninges. Dura-sensitive thalamic neurons that receive direct input from melanopsin-containing photosensitive retinal ganglion cells project to cortical areas involved with pain processing and visual perception. This convergence of retina originating photic pathway onto the trigeminovascular thalamo-cortical pathway may provide an anatomo-pathophysiological substrate for light-induced migraine headache exacerbation [74].

Motivated by the angle-rich visual fortification pattern in the classic migraine visual aura, we used a fMRI-specific paradigm and found that activations in the visual cortex interictally were clearly distinct in people with migraine, indicating that the migrainous occipital cortex is especially responsive to vision of angles. In this study, visual stimuli consisted of 8 rows of 12 parallel oblique (30 degrees inclination) white bright bars on a black background, alternating rhythmically every half second with similar bars orientated 30 degrees to the opposite direction. In the control condition, all bars were parallel to each other. In the main conditions, a column of bars, either on the left or right side, was orientated towards the opposite direction, forming 60-degree angles with the remaining bars [83]. The stimulation threshold to induce phosphenes when transcranial magnetic stimulation is applied over the occipital cortex is lower in patients with migraine than in control participants [84]. Taken together, these data suggest that the visual cortex is relatively hyperexcitable in migraine on a constant basis, and during an attack, light enhances migraine headache severity.

### 7.2. Visual Discomfort

Marcus and colleagues reported that 82% of the people with migraine have an aversion to striped patterns [85]. In fact, a series of visual stimuli such as flicker, glares or stripes can trigger migraine and/or elicit discomfort or aversion [86]. In addition, Shepherd and colleagues reported that patients with migraine experience a greater number of visual illusions for black and white patterns, which may be associated with an imbalance between the excitatory and the inhibitory systems [86]. Patients with migraine report more illusions than control participants, regardless of the size of the stimulus; they also experience more discomfort when viewing the patterns [87,88]

### 7.3. Motion Sickness

The symptoms of motion sickness are related to a conflict between the visual system and the vestibular system. The brainstem also plays an important role in this phenomenon [89]. The incidence of motion sickness is higher in children with migraine (up to 45%, compared with 5% to 7% in children with nonmigraine headache, seizure disorders, or learning disabilities) [90], and a history of motion sickness is predictive of childhood migraine [91]. Later on, people with migraine have greater motion sickness susceptibility [92,93] because more than half are prone to it [94]. Although the exact reasons for increased susceptibility to motion sickness in migraine are not known [89], it is interesting to note that serotonin synthesis reduction (by tryptophan depletion) increases motion sickness and sensitivity to light, possibly through an imbalance between the inhibitory and the excitatory systems [95].

A recently recognized syndrome, persistent postural-perceptual dizziness—PPPD, refers to individuals who present non-spinning vertigo and a sense of unsteadiness [96]. A relationship between PPPD and migraine has been suggested [97]. Interestingly, PPPD can be visually triggered [98], as can motion sickness. Most likely motion sickness and PPPD intermingle. Further observations will cast light on the actual distinction between these two phenomena. People with migraine are also more susceptible than the general population to symptoms evoked by visual stimulation of movement [99,100].

## 8. Visual Stimuli as Migraine Triggers

Environmental trigger studies in migraine are greatly limited by methodological constraints related to patients’ subjective interpretations. Objective, clear-cut stimuli are easier to address and allow firmer conclusions to be drawn. These include visual stimuli, which are recognized as some of the most frequent attack triggers. In addition, migraine-triggering visual stimuli are similar to patterns that trigger seizures, such as flickering/intermittent light and repetitive geometric patterns, or repetitive figures such as stripes [101,102], which is in line with the increased interictal photophobia possibly related to cortical hyperexcitability.

We have examined patients who had attacks induced by stripes, flickering, particular bright colors (e.g., an orange couch, a red wall), and visual patterns (e.g., a curtain with big red roses motifs). A frequent report as a trigger in our patients is driving during the night at a constant speed, staring successive, intermittent sources of light [103]. One of our cases, a young female migraine with aura patient, had attacks with aura triggered specifically by reading. She needed to stop reading every so often to avoid constant migraine attacks. In this patient, patterns having the same light intensity, or a meaningless sequence of random letters would not trigger any aura or headache, suggesting that the reading was the actual trigger factor. The reading discomfort in migraine can also be related to the visual characteristics of the text [86].

Up to 60% of people with migraine vs. 15% of control participants (subjects with headache not fulfilling IHS diagnostic criteria for migraine) report that visual patterns may trigger attacks [104]. Shepherd et al. used a comprehensive approach to study visual processing interictally in 28 migraine (14 migraine with aura, 14 migraine without aura) and 14 headache-free control participants. They assessed visual discomfort (also termed pattern sensitivity or pattern glare) by gauging experiences of discomfort, illusions or distortions while viewing repetitive patterns such as stripes. They also assessed sensitivity to achromatic and chromatic patterns. They included a visual discomfort questionnaire, a hue test, a contrast sensitivity test, a migraine trigger inventory, and a stereopsis test [86]. In their study, MWA patients had more headaches triggered by visual stimuli than control participants. The most frequent triggers were flicker, followed by computer use, stripes, patterns of light and shade, television, cinema, and bright fluorescent pink and green color contrasts. In the MWoA group, computer use or overuse was the most frequent trigger, followed by flicker, patterns of light and shade, stripes, cinema, television, and high contrasts (abrupt transitions from light to dark, driving at night with oncoming car headlights). Visual discomfort was greater among patients, which was reduced by using colored gratings rather than achromatic (black-and-white).

For both patients and clinicians, it is relevant to explore possible visual triggers apart from the more frequently reported sunlight. Migraine aura and without aura are equally affected by possible visual triggers. Geometric patterns, sharp contrasts, colorful or black-and-white may trigger migraine attacks in sensitive subjects.

## 9. Vision and the Neural Underpinnings of the Migraine Related Visual Phenomena

The visual cortex represents a large portion of the brain, as it extends from the occipital cortex to the temporal and parietal cortices. Small lesions in the visual system can readily translate into symptoms such as a loss of part of the visual field. This may be one of the reasons why so many auras have visual manifestations. According to Aristides Leão, who discovered cortical spreading depression (CSD) in 1944 [105], “it seems well established that an essential part of the mechanism of CSD is transmission of a disturbance of cell membrane function, from one cell to its neighbors by diffusion of substances in the extracellular fluid. Therefore, close proximity of the cells certainly facilitates CSD. The density of packing of the cells varies with the region of the cortex, and is by far highest in the visual area. Thus, one would expect this area to be the most liable to suffer from CSD, and in fact visual symptoms are the most frequent in the migraine aura” [106] (p. 20).

In the first paper that related visual cortex organization with visual percept in migraine [107], we noticed that the symptoms experienced by participants with migraine—akin to TV visual snow—corresponded to the type of stimuli that are processed in the area where the origin of the CSD was located, namely V3A [108]. It is very possible, although it has never been demonstrated, that classical fortification spectra may relate to CSD initiating in V1, whereas other types of visual percepts such as flashes of color may start in area V8 [11], or difficulties with face recognition may be associated with activity in the fusiform face area, FFA [109].

Other more cognitively elaborate percepts such as the AIWS micro/macro/metamorphopsias may on the other hand come from associations area such as the parietal/temporal cortices. Interestingly, abnormal perfusion or metabolism were identified in the parietal lobes of patients with visual distortion of size or shape of objects, and in the temporal lobe of a patient with color misrecognition [74].

## 10. Conclusions

Migraine is a common neurological condition associated with visual processing abnormalities and various visual misperceptions across all of its phases, including the interictal phase. Such phenomena encompass rich, sometimes bizarre symptoms much beyond the frequently recognized aura and photophobia complaints. For some patients, migraine affect mostly vision, in the sense that the impact of the visual symptoms is much greater than the headache itself. A better understanding of what triggers their migraine may help them reduce the number of migraine attacks by avoiding these triggers. Most visual disturbances associated with migraine can be justified by increased neural hyperexcitability, as suggested by clinical, physiological and neuroimaging evidence. This is still debate because there may be distinct changes at different stages of the visual system. Therefore, it may be more appropriate to consider VA as dysfunctions in visual processing [110]. Clinicians are encouraged to proactively search, correctly identify, classify and interpret migraine-related visual problems, which may help expand the present knowledge on the disease and optimize migraine care.
